# Anti-IgLON5 disease with distinctive brain MRI findings responding to immunotherapy: A case report

**DOI:** 10.1097/MD.0000000000024384

**Published:** 2021-01-29

**Authors:** Yan Pi, Li-li Zhang, Jing-cheng Li

**Affiliations:** Department of Neurology, Institute of Surgery Research, Daping Hospital, Third Military Medical University (Army Medical University), 10 Changjiangzhilu, Yuzhong District, Chongqing, China.

**Keywords:** anti-IgLON5 disease, brain magnetic resonance imaging, immunotherapy

## Abstract

**Rationale:**

Anti-IgLON5 disease was first described as a progressive antibody-associated encephalopathy, with multiple non-specific clinical symptoms including sleep dysfunction, bulbar symptoms, progressive supranuclear palsy-like syndrome, cognitive impairment, and a variety of movement disorders. This newly discovered disease presents with unremarkable or unspecific brain magnetic resonance imagings (MRI), and have poor responsiveness to immunotherapy.

**Patient concerns:**

In this case, a 37-year-old man presented with 4-day history of gait instability, dysarthria, and oculomotor abnormalities. The initial neurologic examination revealed mild unsteady gait, subtle dysarthria, and left abducent paralysis.

**Diagnosis:**

The patient was diagnosed with anti-IgLON5 disease, based on clinical features and positive anti-IgLON5 antibodies in serum.

**Interventions:**

Initially, the patient was treated with high dosages of methylprednisolone and immunoglobulins.

Outcomes: The symptoms of patient rapidly improved after high-dose intravenous methylprednisolone and immunoglobulins.

**Conclusions:**

In this paper, we report a new case of anti-IgLON5 disease with major symptoms of gait instability, dysarthria, and oculomotor abnormalities, with distinctive brain MRI findings, and responsive to immunotherapy.

## 1 Introduction

Anti-IgLON5 disease was first described as a progressive antibody-associated encephalopathy in 2014 by Sabater et al.^[1]^ Patients develop antibodies against IgLON5, a neuronal cell adhesion protein, whose function stays unclear so far. Anti-IgLON5 antibodies are thought to cause neurodegeneration in specific central nervous system regions. Neuropathological findings revealed a tau phosphorylation and deposition in the hypothalamus and tegmentum of the brainstem.^[2]^ The clinical manifestation of anti-IgLON5 disease seems to be heterogeneous, mostly including sleep dysfunction, bulbar symptoms, progressive supranuclear palsy-like syndrome, cognitive impairment, and a variety of movement disorders. This newly discovered disease presents with unremarkable or unspecific brain MRI, and have poor responsiveness to immunotherapy. This report demonstrates a case with distinctive brain MRI findings and responsive to immunotherapy.

## 2 Case presentation

A 37-year-old man presented with 4 days of gait instability, dysarthria, and oculomotor abnormalities. He experienced slight excessive daytime sleepiness for several days before his admission. There was no history of autoimmunity, cancer, headache, or neck trauma. There was no family history of similar complaints, stroke like episodes, seizures, or early-onset dementia. On admission, the neurologic examination revealed mild unsteady gait, subtle dysarthria, and left abducent paralysis. In addition, although the patient was able to walk alone, he felt unsteady with a subjective feeling of lateropulsion. The rest of his examination was normal.

**Figure 1:**
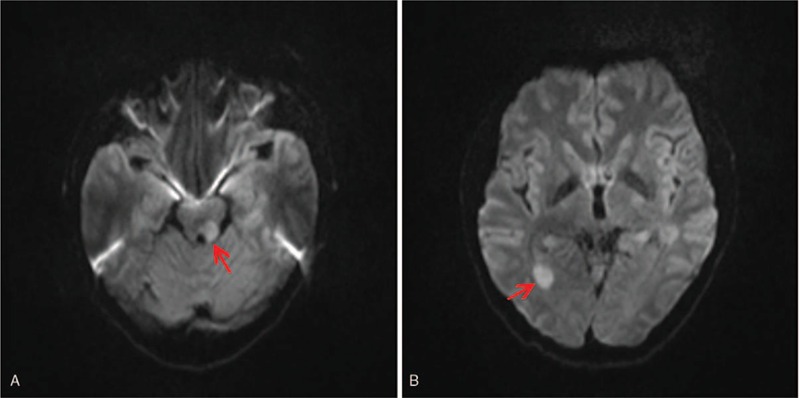
Brain diffusion-weighted magnetic resonance imaging (MRI-DWI) of the anti-IgLON5 disease. Representative axial, MRI-DWI showed asymmetrical areas of reduced diffusion involving left tegmentum of the midbrain (A; arrowhead) and right occipital horn of the lateral ventricle (B; arrowhead).

**Figure 2:**
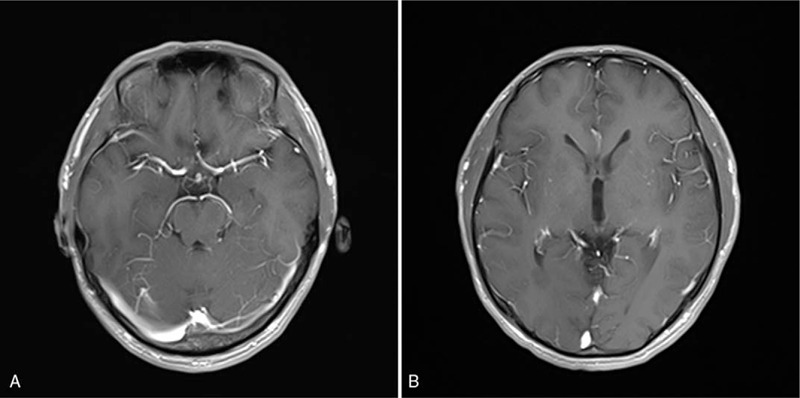
Brain T1-weighted postcontrast MRI of anti-IgLON5 disease. Representative axial, T1-weighted postcontrast MRI demonstrating no significant postcontrast enhancement of left tegmentum of the midbrain (A) and right occipital horn of the lateral ventricle (B).

During a 2-week stay in the department of neurology, MRI of the head and neck, blood, cerebrospinal fluid (CSF), electroencephalogram (EEG), and polysomnogram (PSG) were performed. Two abnormal results were found as follows. The brain MRI showed multiple, scattered diffusion restriction in the bilateral cerebral hemispheres involving left tegmentum of the midbrain (Fig. 1A), and occipital horn of right lateral ventricle (Fig. 1B), without contrast enhancement (Fig. 2A and B). Lumbar puncture was performed. Opening pressure was normal. CSF was clear, and no pleocytosis was detected. CSF analyses showed normal protein with normal CSF/serum glucose ratio. Gram stain of the CSF was unremarkable, and CSF culture remained sterile. Mycobacterium tuberculosis polymerase chain reaction (PCR) was also negative. PCR and enzyme-linked immunosorbent assay tests for arboviruses were also negative. CSF examination results revealed positive oligoclonal bands. Both serum and CSF autoimmune encephalopathy panels were detected, and serologic testing for anti-IgLON5 antibodies returned positive with titer 1:32, while other autoantibodies (Hu, Yo, Ri, CV2, Ma2/Ta, amphiphysin, N-methyl-D-aspartate receptor, α-amino-3-hydroxy-5-methyl-4-isoxazolepropionic acid receptor, contactin-associated protein-like 2, leucine-rich glioma inactivated protein 1, dipeptidyl-peptidase like protein 6, γ-aminobutyric acid b receptor, aquaporin 4, myelin basic protein, myelin oligodendrocyte glycoprotein, and glial fibrillary acidic protein) remained negative in serum and CSF. Furthermore, his human leukocyte antigen (HLA) genotyping confirmed HLA-DRB1∗11:01 and HLA-DRB1∗15:01, HLA-DQB1∗03:01 and HLA-DQB1∗06:02 alleles, and did not show the same HLA association found in other reported cases.^[1,3–5]^ Finally, the EEG and PSG findings were unremarkable.

The patient was initially treated with high-dose intravenous methylprednisolone (1 g/d for 3 days) and immunoglobulins (0.4 g/kg/d for 5 days), which led to a rapid improvement over a few days. His gait instability, dysarthria, and oculomotor abnormalities completely recovered. The titer of serum anti-IgLON5 antibodies decreased to 1:10 after this treatment, and the initial MRI changes have lessened. After intravenous methylprednisolone and immunoglobulins, the patient discharged, continued treatment with mycophenolate mofetil, and oral steroids were tapered slowly over several months. At present, 1 year after disease onset, the patient remains stable with no new exacerbation.

## 3 Discussion

Anti-IgLON5 disease was first described in 2014 as a progressive antibody-associated encephalopathy by Sabater et al.^[1]^ The symptoms of the anti-IgLON5 disease are very heterogeneous. So far, case series and cases have described various clinical symptoms, the most prominent feature is a distinctive sleep dysfunction including rapid eye movement and non-rapid eye movement sleep parasomnia, obstructive sleep apnea syndrome, and excessive daytime sleepiness. Apart from sleep disorders, the most frequent symptoms at disease presentation are gait instability (imbalance and ataxia), bulbar dysfunction (dysphagia, dysarthria), movement disorders (stiffness, dystonia, chorea). Additional, other reported symptoms include oculomotor abnormalities (nystagmus, gaze palsy, slowed saccadic pursuit eye movements), cognitive impairment, autonomic dysfunction (orthostatic dysregulation, sexual dysfunction, bradycardia, perspiration), and symptoms of the peripheral nervous system.^[6]^ These symptoms may each occur in different severity, combinations and periods, leading to various clinical subtypes. Besides the typical clinical symptoms, detection of the antibodies is significant for diagnosis of anti-IgLON5 disease. Antibodies can be detected in both serum and/or CSF.^[6]^ Anti-IgLON5 antibodies are thought to cause neurodegeneration in specific central nervous system regions, which predominantly involves tegmentum of the brainstem, hypothalamus, and hippocampus.^[4]^ Moreover, neuropathological findings revealed a tau phosphorylation and deposition in these regions. Reportedly, more than 95% cases presented unremarkable or unspecific MRI findings, with exceptional cases of mild to moderate brainstem, bilateral hippocampal, and cerebellar atrophy.^[6]^ Of note, most of changes found on MRI are in regions shown affected by the tauopathy. Overall, majority of patients diagnosed with anti-IgLON5 disease were treated with immunotherapy, mostly in combination of at least 2 different therapy strategies, including intravenous corticosteroids, intravenous immunoglobulins, plasma exchange, rituximab, cyclophosphamide, azathioprine, mycophenolate mofetil. However, the clinical response remains poor and the mortality continues to be high.^[6,7]^

In contrast, our case showed distinctive brain MRI changes, particularly in left tegmentum of the midbrain, and occipital horn of right lateral ventricle, which were partly aligned with the regions of neuropathological tau deposition observed in patients with IgLON5 antibodies. After the treatment of corticosteroids and immunoglobulins, his symptoms improved dramatically, his anti-IgLON5 serum titer decreased from 1:32 to 1:10, and his brain MRI showed a reduced range of diffusion restriction in the left tegmentum of the midbrain, and occipital horn of right lateral ventricle. All this suggests that his symptoms could be associated with the titer of anti-IgLON5 antibodies, and the antibodies may play a crucial role in the pathogenesis. Additionally, the positive treatment response to immunotherapy in this patient might also be related to the young age of onset (37 years old) and the only short duration of disease (4 days), while the youngest patient reported in the literature was 40 years old^[3]^ and the shortest duration of disease was 3 months. In this scenario, an early diagnosis and treatment would be crucial to delay or even stop the progress of the assumed pathomechanism. A prompt intervention with immunotherapy could alter the outcome of the anti-IgLON5 disease.

## Acknowledgments

The authors would like to thank the patient for his participation in this study.

## Author contributions

YP: report concept and design and drafting of the manuscript. JCL: report concept and design and editing of the manuscript for critical intellectual content. LLZ: acquisition of data, interpretation of data, critical revision of final manuscript for intellectual content.

**Conceptualization:** Jing-cheng Li.

**Data curation:** Yan Pi, Li-li Zhang.

**Formal analysis:** Yan Pi, Li-li Zhang.

**Funding acquisition:** Yan Pi.

**Investigation:** Li-li Zhang, Jing-cheng Li.

**Writing – original draft:** Yan Pi.

**Writing – review & editing:** Jing-cheng Li.

## Abbreviations

Abbreviations: CSF = cerebrospinal fluid, EEG = electroencephalogram, HLA = human leukocyte antigen, MRI = magnetic resonance imaging, PCR = polymerase chain reaction, PSG = polysomnogram.
